# Ampullary Adenocarcinoma: A Review of the Mutational Landscape and Implications for Treatment

**DOI:** 10.3390/cancers15245772

**Published:** 2023-12-09

**Authors:** Vasileios Tsagkalidis, Russell C. Langan, Brett L. Ecker

**Affiliations:** 1 Division of Surgical Oncology, Rutgers Cancer Institute of New Jersey, New Brunswick, NJ 08901, USA; vt308@cinj.rutgers.edu (V.T.); russell.langan@rwjbh.org (R.C.L.); 2 Rutgers Robert Wood Johnson Medical School, New Brunswick, NJ 08901, USA

**Keywords:** ampullary, adenocarcinoma, periampullary, mutations, targeted therapy

## Abstract

**Simple Summary:**

Ampullary tumors are rare malignancies of the upper gastrointestinal tract. Histologic, immunohistochemical and, most recently, genomic biomarkers have been used to improve prognostication and identify patient subsets most likely to benefit from systemic therapies. Contemporary genomic evaluation has unveiled altered genes and cellular signaling pathways, with potential implications for precision oncology. This review provides a summary of the literature regarding ampullary adenocarcinoma, and outlines current and future directions to improve the oncologic treatment of this disease.

**Abstract:**

Ampullary carcinomas represent less than 1% of all gastrointestinal malignancies with an incidence of approximately 6 cases per 1 million. Histologic examination and immunohistochemistry have been traditionally used to categorize ampullary tumors into intestinal, pancreatobiliary or mixed subtypes. Intestinal-subtype tumors may exhibit improved survival versus the pancreatobiliary subtype, although studies on the prognostic value of immunomorphologic classification have been inconsistent. Genomic classifiers hold the promise of greater reliability, while providing potential targets for precision oncology. Multi-institutional collaboration will be necessary to better understand how molecular classification can guide type and sequencing of multimodality therapy.

## 1. Introduction

Ampullary adenocarcinomas are rare malignant epithelial tumors of the ampulla of Vater. Ampullary neoplasms represent 0.2% of all GI malignancies, 4–16% of all periampullary carcinomas and 9–15% of all pancreatoduodenectomies [1,2,3,4,5,6,7]. According to the surveillance, epidemiology and end results (SEER) registry, ampullary adenocarcinoma has an annual incidence rate of approximately 6 cases per 1 million [8,9].

Ampullary tumors are part of a larger group of neoplasms called periampullary neoplasms. Periampullary neoplasms include tumors that arise within the ampulla and its adjacent structures, including the duodenum, intrapancreatic common bile duct and pancreatic head [1,10]. It can be clinically and pathologically difficult to identify the exact origin of a periampullary tumor, especially in the setting of tumor infiltration. While surgical treatment (i.e., pancreatoduodenectomy) is the same for these malignant tumors, the long-term outcomes between adenocarcinomas of the periampullary region vary significantly. Over 80% of ampullary carcinomas are resectable, with 5-year survival rates reaching over 30–50%, which is significantly higher than the rates of resection and survival observed in cholangiocarcinoma and pancreatic cancer [3,6,11,12,13,14,15,16]. However, despite the higher resectability rates of ampullary adenocarcinomas, the majority of patients with resected ampullary carcinoma will still succumb to their disease [17]. Molecular diagnostics may have a role in improving prognostication and treatment.

## 2. Anatomy of the Ampullary Region

The ampullary region is the crossroad of three different structures: the bile duct, the main pancreatic duct (of Wirsung), and the duodenal mucosa. Often, the distal bile duct joins the pancreatic duct within the duodenal wall, forming a common channel (usually <3 mm in length), before emptying into the duodenal lumen through the orifice of the papilla at the ampulla (hepatopancreatic ampulla). Smooth muscle fibers cover the intra-duodenal parts of the distal common bile, pancreatic duct and common channel and regulate flow. Occasionally, the distal bile duct and main pancreatic duct can drain separately through different papillary orifices, or drain through a common ampullary orifice but contain an inter-ductal septum. Additionally, the distal bile duct can join the main pancreatic duct before entering the duodenal wall, thus, forming a long common channel [18,19]. Of note, the extra-duodenal confluence of the two ducts is considered a congenital anomaly and has been associated with the presence of choledochal cysts and gallbladder cancer, possibly due to reflux of pancreatic effluent into the common bile duct [20].

According to WHO classification, ampullary tumors have their epicenter in the ampullary region. This is determined during the gross examination of the resected specimen using the bivalving approach, during which the pancreatic duct and bile duct are sectioned in the same plane along their course towards the ampulla [1]. Similarly, in contemporary pathology texts, a tumor is defined as ampullary if >75% of the tumor is localized in the ampulla or engulfed by it [18]. The College of American Pathologists (CAP) defines three types of tumors based on location; intra-ampullary, when the tumor arises in the ampulla; peri-ampullary, when it arises from the duodenal surface of the papilla; and mixed-type when it involves both regions. Intra-ampullary tumors can arise in the setting of a precursor lesion, called intra-ampullary papillary-tubular neoplasm (IAPN), or from the ampullary ducts [18,21].

## 3. Histology

As discussed earlier, three major structures with distinct histology converge in the ampullary region. The duodenal surface of the papilla of Vater is lined by small intestinal epithelium. As the intestinal epithelium reaches the edge of the papilla, it transitions to mixed epithelium with mucinous features and goblet cells, resembling gastric-foveolar epithelium. The wall of the ampulla consists of pancreatobiliary-type ductules that are nested within the muscle fibers of the sphincter of Oddi. Similarly, the distal bile and pancreatic duct contain small pancreatobiliary-type tributaries and peribiliary mucous glands [9,18,22]. 

Ampullary adenocarcinomas can have histologic features that mirror these distinct histologic origins. Kimura et al. was first to histologically dichotomize ampullary carcinomas into intestinal (INT) and pancreatobiliary (PB) subtypes [23]. INT-ampullary tumors resemble colonic adenocarcinomas and are thought to arise from a preexisting adenoma. They form tubular glands and solid nests composed of tall columnar cells with elongated hyperchromatic and pseudostratified nuclei. Goblet cells can be interspersed with the columnar cells. These tumors usually stain negative for mucin 1 (MUC1) and positive for either cytokeratin 20 (CK20), caudal-type homeobox 2 (CDX2) or mucin 2 (MUC2). They can also stain positive for all three, CK20, CDX2, MUC2, irrespective of MUC1 staining. PB tumors can resemble pancreatic ductal adenocarcinomas. They consist of cuboidal or low columnar cells with round centrally placed nuclei arranged in a single layer, that form small glands in desmoplastic stroma. They exhibit MUC1 positivity and MUC2, CDX2 negativity, while they can stain positive or negative for CK20 (Table 1). Mixed-type tumors lack specific features that would categorize them as purely intestinal- or pancreatobiliary-type tumors; however, a predominant pattern should be documented by the pathologist [1,21,22,24,25]. 

While immuhistochemistry (IHC) and hematoxylin-eosin (H&E) staining can be helpful in determining the tumor suptype, their use is limited by the subjective interpretation of these results by the pathologists, staining thresholds, and overlapping features between subtypes [22]. In a study published by Ang et al., interobserver variability between pathologists and the added value of IHC staining to differentiate between subtypes was evaluated. Using H&E staining alone, an agreement in histologic classification (defined as an agreement in ≥3 out of 4 pathologists) was achieved in 77% of specimens, although this was driven primarily by higher kappa (κ) scores for the mucinous and poorly differentiated subtypes (κ = 0.89 and 0.72, respectively; excellent agreement). In contrast, there was an inferior agreement for the intestinal pancreatobiliary and mixed types (κ = 0.57, 0.48: good agreement; 0.09: poor agreement, respectively). When IHC was used, 83% of the cases that had discordant H&E diagnoses among pathologists were classified as either INT or PB. Overall, when both H&E and IHC were used, 92% of specimens were able to be classified as INT or PB. Notably, the majority of mucinous types diagnosed by histology were classified as INT by IHC (n = 7/8), and over two thirds of mixed (n = 9/13) and poorly differentiated (n = 9/12) (defined as carcinomas with infiltrating single cells or solid pattern without specific INT/PB morphology) tumors classified by H&E were reclassified as either INT (n = 7) or PB (n = 11) with IHC [25]. In validation, a French study revealed a κ score of 0.815 between pathologists when histologic and IHC classification was used [26].

## 4. Prognosis Based on Histologic Subtype

The identification of distinct histologic or molecular subtypes is only relevant if such subtypes define patient subsets with unique prognosis or response to therapy. Early publications suggested that histology might provide such a prognostic biomarker. In 2013, Chang et al. analyzed 72 resected ampullary adenocarcinoma patients. The authors observed improved median survival associated with INT tumor type (115.5 months vs. PB: 16 months; *p* < 0.001) [13]. In an independent study cohort of 95 ampullary adenocarcinomas, INT subtype tumors evidenced improved median survival compared to PB subtype tumors (98 months vs. 25 months, *p* < 0.001) [27]. Similar findings have been confirmed in at least three additional studies [28,29,30]. 

However, the markedly poor prognosis observed for resected PB subtype tumors has not been confirmed in several external studies, leading to a lack of association between histomorphologic subtype and survival [12,31,32,33,34,35]. The European Study Group for Pancreatic Cancer (ESPAC)-3 randomized controlled trial (RCT), which included 428 patients with resected non-pancreatic periampullary tumors, where notably, 69% had ampullary adenocarcinomas, represents the largest cohort of ampullary adenocarcinoma patients studied in the contextual rigor of a phase III trial. Here, there was no difference in the overall survival between INT and PB subtypes [12]. Similarly, Moekotte et al. published one of the largest cohorts (n = 887) of resected ampullary adenocarcinoma where histomorphologic subtype was not prognostic of long-term survival. In this retrospective multicenter study, 547 patients had data on histologic subtypes. Fifty-four percent were of PB-subtype, followed by INT (38%) and mixed (8%). A histologic subtype was not associated with survival in a multivariate Cox regression (*p* = 0.175) [36]. Additionally, several institutional analyses have failed to identify a statistically significant difference in survival between the two subtypes in resected patients [37,38]. Ultimately, histomorphologic subtypes provide simple categories for classification, but the utility of such a biomarker in guiding post-surgical surveillance or treatment decisions is limited given this variability.

## 5. Systemic Therapies

While patients with an ampullary adenoma with or without dysplasia can be managed with an endoscopic resection, the presence of a localized carcinoma requires oncologic resection with pancreatoduodenectomy [22]. Still, the high rate of recurrence compels a multimodality approach in the majority of patients.

Neoadjuvant therapy: There is a lack of strong scientific evidence to support the use of neoadjuvant therapy in ampullary adenocarcinoma. The role of perioperative chemotherapy for related pancreatic adenocarcinomas is still being defined by ongoing trials, and such data may inform future strategies in the management of resectable ampullary adenocarcinoma. If neoadjuvant therapy is considered, such a decision should be made within the scope of a multidisciplinary discussion. 

Adjuvant therapy: Following resection, the National Comprehensive Cancer Network (NCCN) guidelines endorse either observation (AJCC Stage I and II) or adjuvant systemic therapy (AJCC Stages I, II, or III) with or without chemoradiation [22]. Given the rarity of this disease, there is a lack of strong scientific evidence to guide the use of systemic or locoregional therapies. Data available for interpretation are often derived from retrospective analyses of institutional or national databases, or randomized trials where ampullary tumors represent a subset of other periampullary tumors [15,39]. To date, at least four RCTs have assessed the impact of adjuvant therapy in resected periampullary carcinoma [12,40,41,42]. Only the Japanese Study Group of Surgical Adjuvant Therapy for Carcinomas of the Pancreas and Biliary Tract RCT, where no survival benefit was observed for the adjuvant mitomycin C and 5-fluorouracil (5-FU) relative to surgery alone, included a subset analysis of ampullary adenocarcinoma patients [40]. The ESPAC-3 RCT, as mentioned above, included randomization to adjuvant chemotherapy with either 5FU/LV or gemcitabine versus observation. Patients with ampullary cancer who received gemcitabine had a median survival of 70.8 months, versus 57.8 months and 40.6 months in the 5FU/LV and observation groups, respectively—although no statistical comparison between the treatment arms was provided. In bivariate regression analysis for all cancer types (i.e., not limited to the ampullary adenocarcinoma subset), neither treatment improved survival. In contrast, in a multivariable Cox model, there was an improvement in survival for patients receiving adjuvant chemotherapy compared to observation (HR 0.75, 95% CI 0.57–0.98) and for use of gemcitabine compared to observation (HR 0.70, 95% CI 0.51–0.97) [12]. 

Locally advanced, metastatic or recurrent disease: Given the limited randomized data and the histologic similarity of ampullary adenocarcinomas with other related malignancies of the intestine, pancreas and bile duct, current NCCN recommendations are extrapolated from pancreatic, colorectal and biliary tract trials for these treatment regimens. Treatment of recurrent disease after curative-intent resection depends, in part, on previous adjuvant treatments, disease-free intervals, disease extent and patient performance status. Disease recurrence develops locoregionally (11–15%) and/or distally, with the most common sites being the liver (10–67%), lungs, bone and peritoneum. Lymphovascular or perineural invasion, lymph node involvement, and increasing T-stage have been reported as factors associated with increased risk of recurrence following resection [43,44,45]. Systemic chemotherapy remains as the main treatment option for patients with recurrent disease, with locoregional therapies considered on a case-by-case basis for patients with oligometastatic disease or for symptom palliation [22,43]. 

## 6. Histologic Subtype to Guide Adjuvant Treatment Selection

NCCN guidelines recommended consideration of histologic subtypes when choosing systemic chemotherapy. For INT-subtype tumors, guidelines recommend applying the same chemotherapeutic regimens used for colorectal cancer. For PB and mixed subtypes, the guidelines endorse systemic options offered for hepatocellular carcinoma, biliary tract malignancies and pancreatic adenocarcinoma [22]. However, the histologic subtype is not a reliable prognostic biomarker, and the data to support its use as a predictive biomarker is even more limited. The ESPAC-3 trial, detailed above, evaluated whether INT vs. PB subtype differentiated response to adjuvant systemic therapy. The authors report that the histologic subtype was not a predictor of response to treatment, although these data are not directly provided. In previous work from our group, efficacy of adjuvant chemotherapy was evaluated with stratification by histologic subtypes and chemotherapeutic agents. In a multi-institutional series of 357 patients with resected ampullary adenocarcinoma, neither the administration of gemcitabine-based nor fluorouracil-based chemotherapy was associated with improved survival for either the INT or PB subtype [31]. Of note, the role of histologic subtypes to guide systemic treatment selection for other contexts (i.e., neoadjuvant therapy or first-line metastatic therapy) have not been previously studied.

## 7. Genomic Mutations

Because of the limited and conflicting data regarding the response of ampullary tumors to adjuvant therapies, studies have attempted to identify genomic subtypes that may help guide treatment and improve prognostication. 

Several gene mutations in major cell-signaling pathways have been identified. Those pathways are the WNT pathway, the RTK/RAS/MAPK/PI3K pathway, activin/TGF beta (β) signaling pathway, p53 pathway, and chromatin remodeling (Table 2) [46,47]. 

In 2016, Yachida et al. performed whole genome sequencing and copy number analysis of 60 ampullary adenocarcinomas followed by targeted genomic analysis of another 112 ampullary carcinomas, for a total of 172 tumors. A total of 24 genes with significant mutations were identified. INT tumors (n = 93) had frequent mutations in APC (49.5%), TP53 (46.2%), KRAS (38.7%), CTNNB1 (25.8%), ERBB2 (14%), ACVR2A (12.9%), SMAD4 (12.9%), GNAS (12.9%), SOX9 (12.9%), LOXHD1 (12.9%), ELF3 (11.8%), ACVR1B (10.8%), BRAF (10.8%), amongst others. PB tumors (n = 66) had significant mutations in KRAS (68.2%), TP53 (66.7%), SMAD4 (19.7%), CTNNB1 (15.2%), APC (13.6%), ERBB3 (13.6%), GNAS (12.1%), CDH10 (12.1%) and ELF3 (10.6%). Mixed-type tumors (n = 13) had mutations in TP53 (69.2%), CTNNB1 (46.1%), SMAD4 (23%), APC (23%), ELF3 (23%), ARID2 (23%), ERBB2 (15.3%), and CNTN4 (15.3%) (Table 2).

INT tumors had more frequent alterations in the WNT signaling pathway (76%) compared to PB tumors (38%) (*p* < 0.001). In contrast, PB tumors had more frequent alterations in RTK-RAS (81.8%) and P53-Rb (74.2%) pathways (versus 63.4%, *p* = 0.006 and 54.8%, *p* = 0.008; respectively). A significant number of patients were found to have a mutation in ELF3, a tumor suppressor gene. To assess its function, the authors used small interfering RNAs to knockdown the ELF3 in normal bile duct and duodenal mucosa epithelial cells. This knockdown was found to be associated with epithelial–mesenchymal transition, with upregulation of ZEB1, ZEB2 and TWIST1, as well as increased motility and invasion [24]. 

In another study, Gingras et al. analyzed the exome sequence and copy number variations of 98 ampullary, 44 distal bile duct and 18 duodenal adenocarcinomas. WNT pathway alterations varied by tumor type, where it was most frequently observed in duodenal adenocarcinomas (72%), followed by the ampullary (49%) and distal bile duct (30%) carcinomas. SMAD4, a gene altered in up to 60% of pancreatic ductal adenocarcinomas, was found to be to the most commonly mutated gene in the TGF-β pathway within the ampullary and bile duct carcinoma groups. KRAS, another gene frequently mutated in pancreatic cancer, was commonly found mutated within all three groups. Multivariate analysis showed that mutations in the PI3K and TGF-β signaling pathways were associated with improved OS, with HR 0.43 (*p* = 0.036) and HR 0.42 (*p* = 0.005), respectively. Similarly to Yachida et al., the authors identified a significant portion of ELF3 gene mutations within the TGF-β signaling pathway [46,48]. 

A French Association des Gastro-Entérologues Oncologues (AGEO) study used next-generation sequencing to analyze the genomic profile of 91 ampullary adenocarcinomas. The authors found that KRAS mutations were present in almost half of the tumors (n = 41, 45.1%), with the majority in codon 12 (n = 35). Other mutations identified were TP53 (n = 35; 38%), APC (n = 14; 15%), PIK3CA (n = 12; 14%), SMAD4 (n = 8; 9%) and, less frequently BRAF (n = 7; 8%), CDKN2A (n = 4; 4%), FBXW7 (n = 4; 4%), ERBB2 (n = 2; 2%), PDFGRA (n = 2), SMARCB1 (n = 2), RB1 (n = 2). Approximately two thirds of the PB tumors and one-third of the INT tumors had detectable KRAS mutation, a significant difference irrespective of tumor classification by histology (*p* < 0.04) or IHC (*p* < 0.02) [26].

Lastly, Mikhitarian et al. analyzed 52 patients with ampullary adenocarcinoma. In total, 48% were INT and 46% were PB type. One quarter of the tumors were found to express epidermal growth factor receptor (EGFR), with the majority of those being PB (n = 10/24) and only one tumor being INT (*p* = 0 = 002). Almost half of the tumors had a KRAS mutation (n = 25/52). No statistically significant difference was identified between the number of INT and PB tumors that harbored a KRAS mutation (n = 13 vs. n = 10, *p* = 0.57). Additionally, no significant difference in survival was found between tumors with KRAS codon 12/codon 13 mutation and wild-type (*p* = 0.811) [35]. Among the KRAS-mutant tumors, five tumors had additional mutations in SMAD4 and/or PIK3CA. SMAD4 and PIK3CA exon 9 mutations were detected in three and six tumors, respectively. Half of the PB tumors (n = 12) had a detectable mutation in either KRAS (codon 12, n = 7; codon 61, n = 3), PIK3CA (codon 545, n = 1) and BRAF (codon 600, n = 1). Majority of INT tumors (n = 15/25) had an identifiable mutation in KRAS only (codon 12, n = 8/25), PIK3CA (codon 545) only (n = 2/25) or KRAS + PIK3CA and/or SMAD4 (n = 5/25) [35].

In an attempt to move from individual gene alterations to a genomic classifier, our group employed a hidden-genome model to create a molecular taxonomy for ampullary adenocarcinoma. The hidden-genome model is a statistical approach that aggregates both common and rare genomic variants in a multilevel meta-feature regression to predict the cell of origin for a cancer sample [49,50]. While frequent and highly conserved variants comprise a small number of observed alterations detailed above (e.g., alterations in WNT and RTK-RAS pathways), deep sequencing has revealed millions of unique somatic mutations, and often, >90% of somatic variants are singletons. The hidden genome classifier incorporates both common and rare variants to optimally determine the cancer subtype. In our published work, the ampullary hidden-genome model was trained on a prospectively sequenced cohort of 3,411 patients with related malignancies of the pancreas, distal bile duct and large intestine. The model was subsequently applied to targeted panel DNA sequencing data from 76 ampullary adenocarcinomas. The colorectal genomic subtype prediction was primarily influenced by mutations in APC and PIK3CA, tumor mutational burden, and DNA mismatch repair (MMR) deficiency signature. Pancreatic genomic subtype prediction was dictated by *KRAS* gene alterations, particularly *KRAS* G12D, *KRAS* G12R, and *KRAS* G12V. Distal bile duct adenocarcinoma genomic subtype was most influenced by copy number gains in the *MDM2* gene [51]. In an international, six institution validation study of 193 ampullary adenocarcinoma patients, the molecular taxonomy provided unique characterization of the heterogeneity of individual patient tumors and confirmed its prognostic value beyond standard histomolecular classification. No difference in OS was observed between INT and PB tumors classified by histology and IHC. In contrast, genomic profiles consistent with the colorectal cell-of-origin were associated with improved long-term survival, both for patients with INT and PB histologic subtypes [52]. Additionally, genomic profiles consistent with pancreas or distal bile duct cell-of-origin were associated with an inferior prognosis, both for INT and PB histologic subtypes. These data provide support for a reproducible molecular biomarker available from routine clinical sequencing for post-resection prognostication that can be used for the next generation of systemic chemotherapies trials.

## 8. Targeted Therapies

Tumor sequencing has identified several targetable alterations for tumor-agnostic therapies, that, to date, have been used for patients with locally advanced, metastatic or recurrent ampullary adenocarcinoma. One example is mismatch-repair deficiency (dMMR)/microsatellite instability (MSI-H)/high tumor mutation burden (TMB-H). Xue et al. analyzed 127 ampullary adenocarcinomas (68% either INT or PB), with 18% (n = 23) demonstrating abnormal MMR staining. The loss of both MLH1/PMS2 was identified in 15 patients, whereas 2 patients had a loss of both MSH2/MSH6 [53]. Similarly, the hidden-genome colorectal genomic profile is characterized, in part, by high TMB [51]. The MMR mechanism is a cellular system that recognizes and repairs mismatched nucleotides during DNA replication or recombination or from external damage. Somatic or germline mutations in the genes responsible for the MMR repair apparatus (i.e., mutL Homologue 1 (MLH1), mutS Homologue 2 (MSH2), mutS Homologue 6 (MSH6) and postmeiotic segregation increased 2 (PMS2)) can lead to a deficient system. Microsatellites are repetitive sequences of genome, usually less than six base pairs, scattered in the DNA. If two or more repeats are altered, the tumor is considered MSI high (MSI-H), whereas MSI low (MSI-L) is a tumor where only one repeat is altered; if no alterations are found, the tumor is considered microsatellite stable (MSS) [54]. Pembrolizumab, a PD-L1 checkpoint inhibitor, is recommended for ampullary cancers that are dMMR, MSI-H or TMB-H (≥10 mut/Mb); similarly, nivolimumab/ipilimumab is recommended for MSI-H, dMMR INT tumors. Patients with recurrent or advanced MSI-H or dMMR disease are candidates for Dostarlimab-gxly, another PD-L1 inhibitor [22]. 

Another molecular target is BRAF V600E, which is altered in up to 9.3% of ampullary adenocarcinomas [24,55]. The BRAF kinase is serine/threonine protein kinase that belongs to the RAF family. It is usually activated by the RAS family in response to receptor tyrosine kinase (RTK) activation. This activates the MEK (MAPK kinase) and mitogen-activated protein kinase (MAPK) (also called extracellular signal-regulated kinases [ERK]) which eventually promotes cell function, differentiation and proliferation. Mutated BRAF from the substitution of glutamic acid (E) for valine (V) at the 600th amino acid in the protein (V600E) leads to 500-fold increased activity, with downstream autonomous activation [56,57]. The FDA has approved dabrafenib (BRAF inhibitor) with trametinib (MEK inhibitor) for such patients [22]. 

The neutropic tropomyosin receptor kinase (NTRK) belongs to a family of transmembrane tyrosine kinases that are encoded by the genes NTRK1, NTRK2, NTRK3. Alterations are observed in up to 2% of ampullary adenocarcinoma patients [58,59,60]. The proteins encoded by those genes (TRKA/B/C, respectively) play an important role in activating PI3 kinase (PI3K) and MAPK pathways. NTRK gene fusion leads to a persistent downstream activation of the pathways, leading to tumorigenesis [61]. Laroctretinib and Entrectinib are potent tropomyosin TRKA/B/C inhibitors, approved for ampullary carcinoma patients with NTRK gene fusion [22]. 

The RET gene codes for an RTK which activates the RTK/RAS/MAPK/PI3K pathway. Gene fusion causes ligand-independent activation of the pathway, which leads to tumor growth and proliferation. Selpercatinib, a RET kinase inhibitor, has been approved for locally advanced or metastatic tumors [22]. 

HER2 (ERBB2) is altered in up to 13% of ampullary adenocarcinomas [60,62]. HER2 targeted therapy has been shown to be beneficial in patients in HER2 positive breast and gastric cancer, amongst others. In the MY PATHWAY basket trial, trastuzumab + pertuzumab led to response rates for patients with pancreatic (22%), biliary (29%), and colorectal (38%) cancers with HER2 amplifications, suggesting it may have efficacy for HER-2 amplified ampullary adenocarcinoma [63]. For now, its use in ampullary cancer is currently not recommended by the NCCN Panel.

EGFR is altered in up to 7% of ampullary adenocarcinomas [64]. Data with anti-EGFR–targeted therapies in ampullary adenocarcinomas are limited. Given the limited role of anti-EGFR therapies in KRAS wild-type small bowel cancers or for right-sided colon cancers, anti-EGFR therapies for KRAS wild-type ampullary adenocarcinomas are not recommended [22]. 

Mutations in BRCA2, BRCA1, and PALB2 have also been reported in the literature [60,65]. Gemcitabine + cisplatin can be considered in patients with these mutations, based on randomized data for pancreatic cancer patients with germline BRCA/PALB2 mutations (Table 3) [66].

## 9. Future Directions and Conclusions

There is growing recognition of the heterogeneous genomic landscape of ampullary adenocarcinoma, which mirrors its variable clinical course. Genomic sequencing is an increasingly available technology that allows more precise tumor classification, which is important not only for understanding tumor biology, but also differences in clinical behavior and potential tailored treatment. To date, these data have provided improved post-resection prognostication. Future efforts will evaluate the predictive capacity of genomic models to optimize systemic chemotherapy use and type. Additionally, novel therapies are needed to expand precision oncology from its limited role in metastatic patients with rare actionable mutations to the broader population with this malignancy.

## Figures and Tables

**Table 1:** Tumor types and their associated IHC staining.

	Immunohistochemistry						
Tumor Type	CK20	CDX2		MUC2			MUC1
Intestinal	+	or	+	or	+	and	−
Intestinal	+	and	+	and	+		+/−
Pancreatobiliary	+/−		−	and	−	and	+

**Table 2:** List of cell-signaling pathways and number of ampullary tumors with an associated gene mutation, shown as percent.

		Yachida et al.			Gingras et al. ^#^	AGEO ^+^	Mikhitarian et al. *
Pathway	Mutation	INT	PB	Mixed	Amp	Amp	Amp
WNT	APC	49.5	13.6	23.1	27	15	
	CTNNB1	25.8	15.2	46.1	13		
	SOX9	12.9	1.5	7.7	7		
	FBXW7	7.5	4.5	0	6	4	
	AXIN1				7		
RTK/RAS/MAPK/ PI3K	KRAS	38.7	68.2	7.7	55	46	48.1
	BRAF	10.8	7.6	7.7		8	2
	ERBB2[HER2]	14	7.5	15.3	6	2	
	ERBB3[HER3]	8.6	13.6	7.7			
	GNAS	12.9	12.1	0	6		
	NF1				10		
	PIK3R1				6		
	PIK3CA				18	14	11.5
	PTEN				9	1	0
Activin/TGF-β	ELF3	11.8	10.6	23.1	12		
	TGFBR1	7.5	6	0			
	TGFBR2	7.5	4.5	0	8		
	ACVR2A	12.9	1.5	0			
	ACVR1B	10.8	6	0	5		
	SMAD3				3		
	SMAD4	12.9	19.7	23.1	20	9	5.8
P53-Rb	CDKN2A	2.2	9.1	0		4	
	ATM				13		
	TP53	46.2	66.7	69.2	58	38	
Chromatin remodeling	ARID1A				11		
	ARID2	18	10.6	23.1	12		
	ARID1B				4		
	SMARCA2				2		
	SMARCA4				7		
	PBRM1				5		

^#^ Includes the 45 INT-subtype, 37 PB-subtype and 16 mixed subtype; ^+^ Includes the 43 INT-subtype, 29 PB-subtype, 18 ambiguous subtype and 1 unclassified; * Includes 52 tumors with 3 patients having non-INT/PB pathology.

**Table 3:** Recommended therapies according to NCCN.

Target	Therapies
BRAF V600E	Dabrafenib + Trametinib
MSI-H or MMR deficient tumors	Nivolumab + ipilimumab Pembrolizumab Dostarlimab-gxly
NTRK	Entrectinib or Laroctretinib
RET	Selpercatinib
HER2	Not recommended
EGFR	Not recommended
BRCA/PALB2	Gemcitabine + Cisplatin
